# Clearance of Senescent Cells by BCL_XL_ ‐PROTAC: A Novel Approach to Treat COPD?

**DOI:** 10.1111/acel.70487

**Published:** 2026-04-14

**Authors:** Justine V. Devulder, Peter S. Fenwick, Ewa Kolosionek, May Al‐Sahaf, Patrizia Viola, Raphael Lemaire, Neetu Razdan, Hiromi Kudo, Anthony Sinadinos, Lina Odqvist, Louise E. Donnelly, Peter J. Barnes

**Affiliations:** ^1^ National Heart and Lung Institute Imperial College London London UK; ^2^ Bioscience COPD/IPF, Research and Early Development Respiratory & Immunology, BioPharmaceuticals R&D, AstraZeneca Gothenburg Sweden; ^3^ Imperial College Healthcare NHS Trust London UK; ^4^ Division of Cancer, Department of Surgery and Cancer Faculty of Medicine, Imperial College London UK; ^5^ Bioscience COPD/IPF, Research and Early Development Respiratory & Immunology, BioPharmaceuticals R&D, AstraZeneca Gaithersburg Maryland USA; ^6^ Section for Pathology, Division of Digestive Diseases, Department of Metabolism, Digestion and Reproduction, Faculty of Medicine Imperial College London London UK

## Abstract

Ageing and cellular senescence significantly contribute to the progression of age‐related diseases, particularly chronic obstructive pulmonary disease (COPD). Cellular senescence refers to the cessation of cell division in response to stress and damage. While senescent cells remain metabolically active, they secrete pro‐inflammatory factors that drive disease progression. Senolytic therapies aim to selectively target and eliminate these senescent cells by inducing their apoptosis. This study examines the senolytic potential of BCL_XL_‐PROTAC, a novel proteolysis‐targeting chimera designed to degrade BCL_XL_, in small airway epithelial cells and fibroblasts from patients with COPD. Treatment of COPD small airway epithelial cells and fibroblasts with BCL_XL_‐PROTAC led to their apoptosis through the activation of caspase 3, along with a reduction in senescence markers such as p21^CIP1^, p16^INK4a^ and senescence‐associated β‐galactosidase. The effects of BCL_XL_‐PROTAC were selective for senescent cells and did not affect non‐COPD cells. The clearance of COPD small airway epithelial cells and fibroblasts by BCL_XL_‐PROTAC was associated with an increase in the proliferation marker Ki67 and enhanced cell proliferation. Additionally, in precision‐cut lung slices obtained from COPD patients, BCL_XL_‐PROTAC significantly reduced p21^CIP1^ expression in the airway epithelium, validating its effectiveness in a more complex tissue environment. These findings demonstrate that BCL_XL_‐PROTAC is a potent and selective senolytic agent that may promote lung cell rejuvenation, supporting its potential as a novel therapeutic strategy for age‐related diseases, including COPD.

## Graphical abstract

Cellular senescence plays a significant role in the progression of age‐related diseases like chronic obstructive pulmonary disease (COPD). Our research shows that clearing senescent cells with the senolytic BCL_XL_‐PROTAC reduces senescence markers in COPD airway cells and promotes lung regeneration, suggesting its potential as a novel COPD therapy.

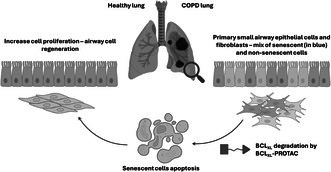

## 1 Introduction

By 2030, one in six people globally will be aged 60 or over (World Health Organization 2024) and this is associated with healthcare challenges that come with an ageing population. Ageing is the time‐dependent accumulation of molecular and cellular damage, leading to gradual decline in physical and mental capabilities, which ultimately increases the risk of illness and death (Gianfredi et al. 2025; Ismail et al. 2021). Ageing is a leading contributor to multiple chronic diseases, including chronic obstructive pulmonary disease (COPD) and is characterised by 12 hallmarks that include cellular senescence (Guo et al. 2022; Lopez‐Otin et al. 2023). Cellular senescence is an irreversible state of proliferative arrest in cells after a finite number of proliferation cycles or in response to cell stressors. Senescent cells remain viable, resistant to apoptosis, exhibit high metabolic activities and have distinct morphological and molecular characteristics (Roger et al. 2021). Cellular senescence is not defined by a singular unique marker but by several that differ dependent on cell type and stressor. These markers include cell growth arrest, increased senescence‐associated β‐galactosidase activity (SA‐β‐gal), telomere‐associated DNA damage, and upregulation of cell cycle inhibitors including p53, p16^INK4a^ and p21^CIP1^. Additionally, senescent cells secrete proinflammatory factors collectively known as the senescence‐associated secretory phenotype (SASP), which contribute to low‐grade inflammation of ageing tissues (de Magalhaes 2024). The accumulation of senescent cells promotes ageing by producing SASP and by impairing tissue homeostasis and regeneration (Devulder 2024; Yousefzadeh et al. 2020). Furthermore, cellular senescence has been identified as a leading pathophysiological mechanism in numerous age‐related diseases (Mylonas and O'Loghlen 2022), including COPD. COPD is one of the most prevalent age‐related diseases worldwide affecting 213 million people in 2021 leading to 3 million deaths annually (Christenson et al. 2022; Safiri et al. 2022). COPD is a complex and multifactorial condition that develops following chronic exposure to hazardous compounds, primarily tobacco smoking and burning biomass, and is characterised by airflow limitation, persistent inflammation and irreversible parenchymal lung tissue destruction (Barnes et al. 2015). The initial pathological changes in COPD occur in the small airways (airways < 2 mm) where changes in cell structure and function are similar to those observed in aged lung (Higham et al. 2025; Hogg et al. 2004; Verleden et al. 2021). Senescent cells accumulate in COPD lungs in greater numbers compared to healthy aged‐matched individuals and include alveolar type II cells, fibroblasts, small airway epithelial cells and endothelial cells (Barnes et al. 2019; Paschalaki et al. 2013; Rivas et al. 2022; Wrench et al. 2024). Small airway epithelial cells, which include club cells, basal cells and ciliated cells, exhibit increased expression of p21^CIP1^ and p16^INK4a^, along with elevated SA‐β‐gal activity in individuals with COPD compared with age‐matched healthy individuals (Bateman et al. 2023; Davis and Wypych 2022). Lung fibroblasts are a heterogeneous population, and very little is known about their role in COPD (Barnes 2019). A recent study isolated a population of senescent small airway fibroblasts from lung tissue of COPD. The findings revealed that these fibroblasts exhibited reduced proliferation, increased secretion of factors associated with the senescence‐associated secretory phenotype (SASP), and heightened activity of SA‐β‐gal. Additionally, these fibroblasts expressed genes associated with oxidative stress response, mitochondrial dysfunction and fibrosis (Wrench et al. 2024). Moreover, COPD is characterised by a persistent state of chronic inflammation of the airways that mirrors the profile of the SASP (Kumar et al. 2014).

In the last decade, targeting senescent cells has emerged as a promising strategy for overcoming age‐related disease and achieving healthy ageing (Childs et al. 2015). The selective elimination of senescent cells in a BubR1 progeroid mouse model delayed the onset of age‐related phenotypes and extended the healthspan of these mice (Baker et al. 2016). Major advances have also been made in developing novel drugs designed to effectively target and eliminate senescent cells. These senolytics, target specific proteins including BCL_2_ family members (Power et al. 2023). Indeed, senescent cells are resistant to apoptosis due to increased expression of the antiapoptotic BCL_2_ family proteins which prevent the oligomerisation of proapoptotic BAX and BAK through interaction with their BH3 motif (Martin et al. 2023). In COPD lungs, upregulation of BCL_XL_ has been found in senescent cells and is linked to their resistance to apoptosis (Zhang et al. 2012). BH3 mimetics, such as navitoclax (ABT263), specifically bind to BCL_2_, BCL_XL_ and BCL_W_ to block their activity, thereby inducing apoptosis in these cells (Zhu et al. 2016). A murine model of pulmonary fibrosis induced by bleomycin was also attenuated by the administration of navitoclax (Chang et al. 2016; Kim et al. 2021). However, clinical studies of lymphoid malignancies have raised concerns about the safety of using navitoclax as a monotherapy as a significant proportion of the trial patients (38.5%) experienced thrombocytopenia (de Vos et al. 2021). To address the toxicity associated with navitoclax but still target BCL_XL_, researchers have developed a proteolysis‐targeting chimera (PROTAC). PROTACs are bivalent small molecules that consist of a ligand recognising the target protein linked to a ligand for an E3 ligase. This allows for the recruitment of the target protein, followed by its degradation through ubiquitination. The objective of PROTAC technologies is to lower the total drug exposure and minimise thrombocytopenia by targeting proteins to an E3 ligase that is less expressed in platelets (Khan et al. 2019). While PROTAC technologies has been shown to effectively target BCL_XL_ and BCL_2_ in vitro for murine cancer treatment (Khan et al. 2019; Yang et al. 2025), its impact on age‐related diseases remains largely unexplored. Therefore, we used a selective BCL_XL_‐PROTAC that targets BCL_XL_ to the Cereblon (CRBN) E3 ligase, which is minimally expressed in platelets (He et al. 2020). We hypothesised that BCL_XL_‐PROTAC is an effective senolytic agent capable of clearing senescent cells isolated from the lungs of patients with COPD and promoting their proliferation.

## 2 Results

### 2.1 BCL_XL_ ‐PROTAC Induces Potent Proteasomal Degradation of BCL_XL_ Protein in Human Small Airway Epithelial Cells

**FIGURE 1:**
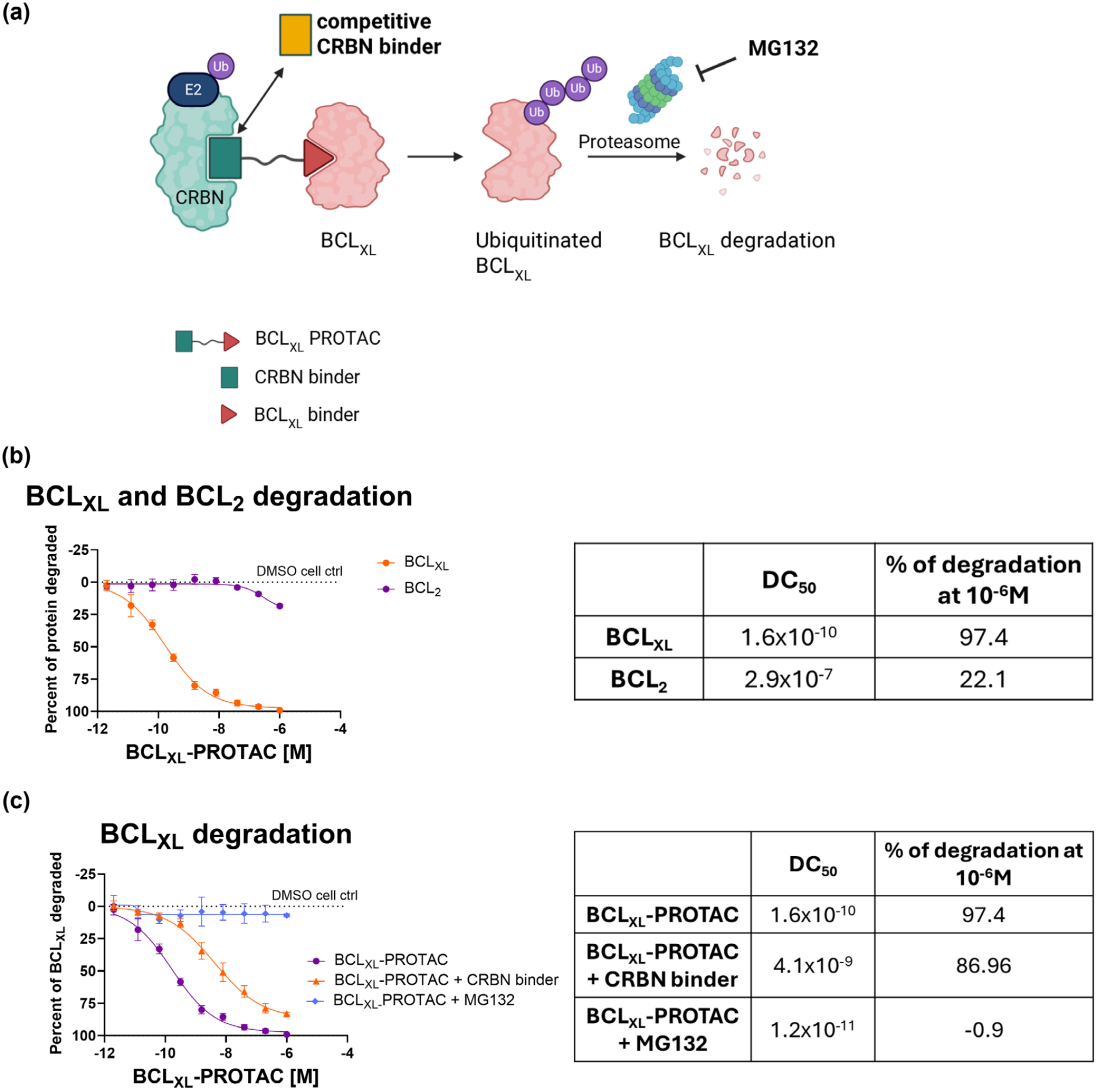
BCL_XL_‐PROTAC induces potent proteasomal degradation of BCL_XL_ protein in human SAECs and shows high selectivity over BCL_2_ degradation. Healthy small airway epithelial cells were treated with 10^−12^ to 10^−4^ M of BCL_XL_‐PROTAC for 24 h. (a) BCL_XL_ degradation by BCL_XL_‐PROTAC via CRBN binder and MG132. (b) Protein levels of BCL_XL_ and BCL_2_ were measured in the cell lysates and the percentage of degradation, and the half maximal degradation concentration (DC_50_) were calculated. (c) Cells were treated with BCL_XL_‐PROTAC and 3 μM of the proteasome inhibitor MG132 or with a CRBN binder to compete with the E3 ligase binding. BCL_XL_ degradation was measured in cell lysates and expressed as percentage of degradation and DC_50_. Plotted data are represented as mean +/− standard deviation of 3 independent experiments, each with 2 technical duplicates. 0% degradation indicate levels detected in vehicle/DMSO‐treated cells, and 100% degradation indicate no detectable BCL_XL_ protein (= medium control).

BCL_XL_ is overexpressed in senescent cells and in COPD small airway epithelial cells (SAEC) which contributes to resistance to apoptosis. In this study, we evaluated whether BCL_XL_‐PROTAC can be used as a senolytic agent through the degradation of its target, BCL_XL_. Healthy SAEC were treated with increasing concentrations of BCL_XL_‐PROTAC for 24 h and protein levels of BCL_XL_ and BCL_2_ were measured in protein lysates (Figure 1a). BCL_XL_‐PROTAC demonstrated a potent and substantial degradation of BCL_XL_ with a half‐maximal degradation concentration (DC_50_) of 0.17 nM and the maximum level of target degradation (Cmax) of 97.4%. The effect of BCL_XL_‐PROTAC was highly selective for BCL_XL_ over BCL_2_ as the DC_50_ for BCL_2_ was 0.29 μM and Cmax was 22.09%. Additionally, inhibiting proteasome activity with MG132 blocked BCL_XL_ degradation, resulting in a Cmax of 0.94% compared to the Cmax of 97.41% for BCL_XL_‐PROTAC alone (Figure 1b). Furthermore, adding a competing CRBN binder to interfere with the binding of the CRBN E3 ligase reduced the potency of BCL_XL_‐PROTAC for BCL_XL_ (Figure 1b). Overall, these results indicate that BCL_XL_‐PROTAC selectively binds to and degrades BCL_XL_ in SAEC, while sparing BCL_2_, through a mechanism that relies on the ubiquitin‐proteasome system.

### 2.2 BCL_XL_ ‐PROTAC Induced Apoptosis and Suppressed Expression of Senescence Markers in COPD SAEC

**FIGURE 2:**
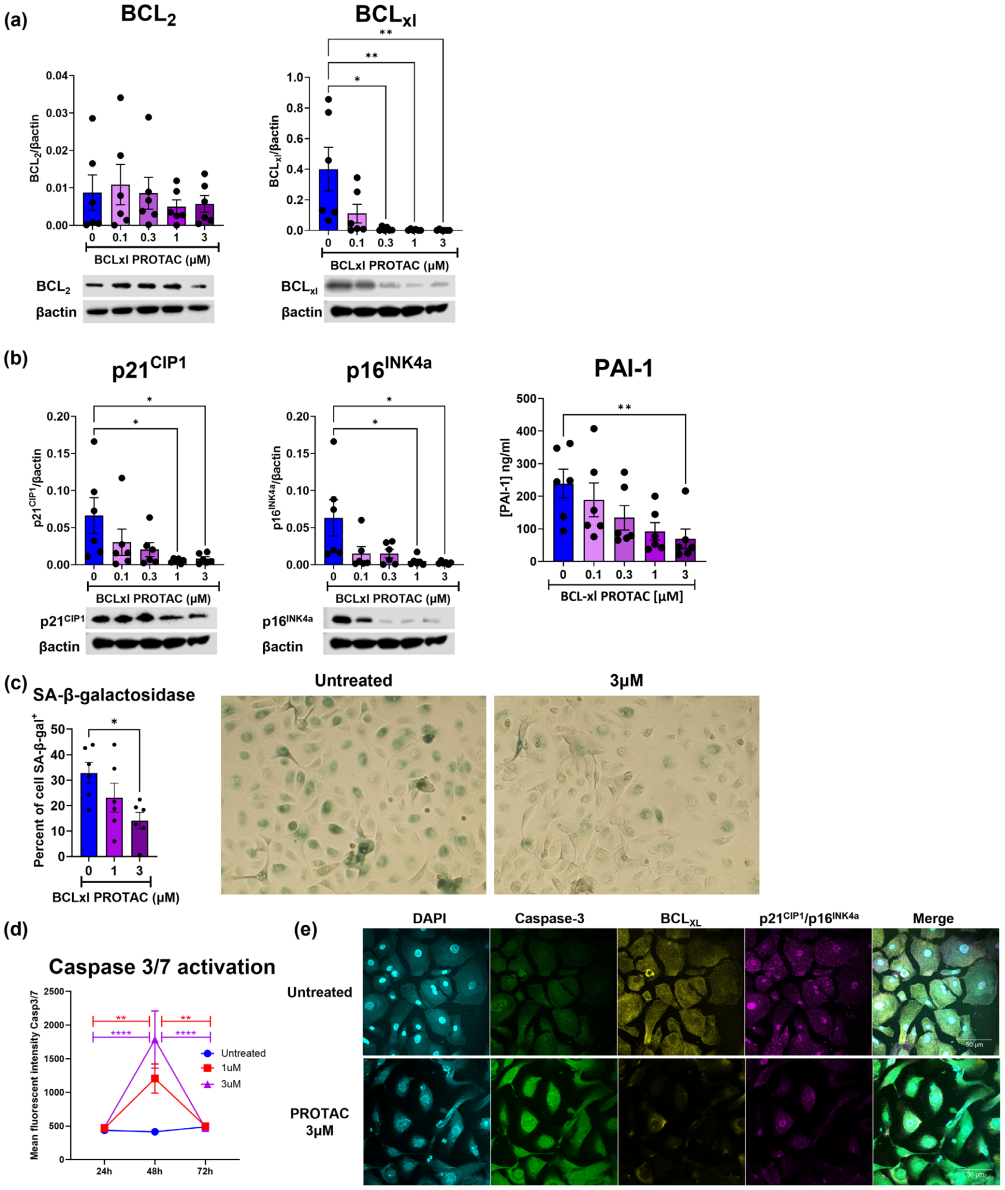
Suppression of BCL_XL_ by BCL_XL_‐PROTAC leads to the decreased expression of senescence markers in COPD SAEC. SAEC derived from COPD lungs were treated with 0.1 μM to 3 μM of BCL_XL_‐PROTAC for 24 to 72 h. (a) After 72 h, the expression of BCL_2_ and BCL_XL_ was measured in cell lysates by Western Blot. (b) The expression of p21^CIP1^ and p16^INK4a^ was measured by Western Blot and the production of the SASP PAI‐1 was measured in cell media by ELISA. (c) After 72 h, COPD SAEC were fixed and stained for senescence associated β‐galactosidase and imaged by light microscopy. (d) After 24 to72 h, the activity of caspase 3/7 was quantified by flow cytometry. (e) After 48 h, cells were fixed and stained for DAPI (turquoise), caspase 3/7 (green), BCL_XL_ (yellow) and p21^CIP1^/p16^INK4a^ (pink) and imaged by fluorescent microscopy. Images are representative of five different experiments conducted on five different patients. Representative Western blots of (a) BCL_XL_ and BCL_2_ and (b) p21^CIP1^ and p16^INK4a^ expression are shown, quantified, and normalised to β‐Actin. Panels (a) and (b) are derived from the same Western blot using the same samples, treatments and timepoint. Data are expressed as mean ± SEM, analysed by Kruskal–Wallis test or Two‐Way ANOVA with post hoc Sidak. *p < 0.05. **p < 0.01. ***p < 0.001. ****p < 0.0001.

Since BCL_XL_‐PROTAC induced degradation of BCL_XL_, but not BCL_2_, we aimed to investigate its effect on COPD SAEC. We treated COPD SAEC with BCL_XL_‐PROTAC for 72 h and assessed the expression of BCL_XL_ and BCL_2_ by Western blotting (Figure 2a). BCL_XL_‐PROTAC did not alter the expression of BCL_2_, whilst BCL_XL_ expression was significantly decreased. Next, we examined whether the degradation of BCL_XL_ affected the senescence phenotype in COPD SAEC by analysing the expression of the senescence markers p21^CIP1^ and p16^INK4a^ in cells treated for 72 h with BCL_XL_‐PROTAC. Both markers showed significant downregulation following treatment with 1 and 3 μM of BCL_XL_‐PROTAC (Figure 2b). Additionally, SASP secretion was also reduced as shown by a significant decrease in PAI‐1 concentration in the cell media of COPD SAEC treated with 3 μM of BCL_XL_‐PROTAC (238.8 ± 44.2 ng/mL in untreated cells vs 69.4 ± 30.05 ng/mL in treated cells). Concentration of CXCL8 and IL‐6 remains unchanged by treatments with BCL_XL_‐PROTAC (Figure S1). To further confirm that treatment with BCL_XL_‐PROTAC leads to a reduction in the senescence phenotype of COPD SAEC, we analysed the activity of SA‐β‐galactosidase (Figure 2c). After 72 h, the percentage of cells positive for β‐galactosidase decreased from 32.8% ± 4.1% in untreated cells to 14.1% ± 3.2% in cells treated with 3 μM of BCL_XL_‐PROTAC. Having demonstrated that degradation of BCL_XL_ results in the downregulation of senescence markers in COPD SAEC, we next investigated whether BCL_XL_‐PROTAC activates caspase 3/7 potentially leading to the apoptosis of senescent cells. COPD SAEC were treated with 1 or 3 μM of BCL_XL_‐PROTAC and activation of caspase 3/7 was analysed by flow cytometry after 24, 48 and 72 h (Figure 2d). The mean fluorescence intensity of activated caspase 3/7 significantly increased after 48 h of treatment with BCL_XL_‐PROTAC compared to both 24 h and 72 h‐timepoints (MFI = 479.3 ± 42 at 24 h for 3 μM of BCL_XL_‐PROTAC vs. MFI = 1785.8 ± 424.9 at 48 h vs. MFI = 468.3 ± 26.9 at 72 h). Thus, caspases are activated 48 h following BCL_XL_‐PROTAC treatment and return to basal levels after 72 h. Fluorescence microscopy further confirmed these results, revealing higher signals of activated caspase 3/7 and a reduction in BCL_XL_‐positive cells after 48 h of treatment (Figure 2e, Figure S2). Altogether, these findings indicate that BCL_XL_‐PROTAC has a senolytic activity where the degradation of BCL_XL_ leads to cell apoptosis via the activation of caspase 3/7 and the downregulation of senescence markers in COPD SAEC.

### 2.3 BCL_XL_ ‐PROTAC Induced Apoptosis and Suppressed Expression of Senescence Markers of a Replicative Senescent‐Enriched Population of Non‐COPD SAEC

**FIGURE 3:**
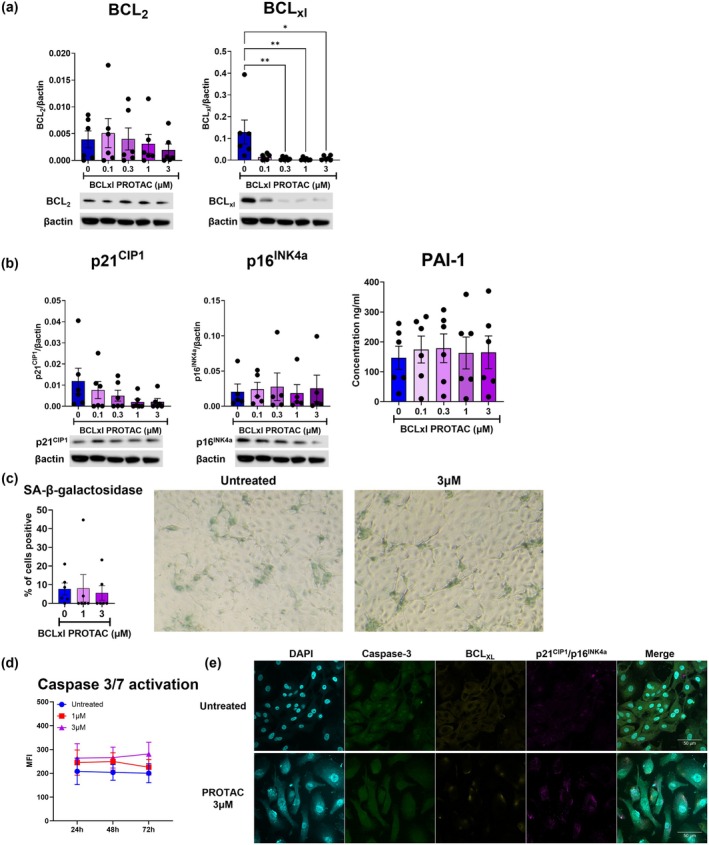
Suppression of BCL_XL_ by BCL_XL_‐PROTAC has no effect on non‐COPD SAEC. SAEC derived from non‐COPD donors were plated and treated with 0.1 μM–3 μM of BCL_XL_‐PROTAC for 24–72 h. (a) After 72 h, the expression of BCL_2_ and BCL_XL_ was measured in cell lysates by Western Blot. (b) The expression of p21^CIP1^ and p16^INK4a^ was measured in healthy SAEC by Western Blot and the production of the SASP PAI‐1 was measured in cell media by ELISA. (c) After 72 h, cells were stained for senescence associated β‐galactosidase and imaged by light microscopy. (d) After 24 to 72 h, the activity of caspase 3/7 was quantified by flow cytometry. (e) After 48 h, cells were fixed and stained for DAPI (turquoise), caspase 3/7 (green), BCL_XL_ (yellow) and p21^CIP1^/p16^INK4a^ (pink) and imaged by fluorescent microscopy. Images are representative of five different experiments conducted on five different patients. Representative Western blots of (a) BCL_XL_ and BCL_2_ and (b) p21^CIP1^ and p16^INK4a^ expression are shown, quantified, and normalised to β‐Actin. Panels (a) and (b) are derived from the same Western blot using the same samples, treatments and timepoint. Data are expressed as mean ± SEM, analysed by Kruskal–Wallis test or Two‐Way ANOVA with post hoc Sidak. *p < 0.05. **p < 0.01.

Having shown that degradation of BCL_XL_ with BCL_XL_‐PROTAC induced apoptosis and reduced expression of senescence markers in COPD SAEC, we next determined whether BCL_XL_‐PROTAC effectively and specifically cleared senescent cells within the mixed population of COPD SAEC. To do this, we cultured SAEC derived from non‐COPD lung tissue for 2 weeks, until they entered a state of ‘replicative’ senescence. These cells showed increased β‐galactosidase activity as well as PAI‐1 and IL‐6 secretion compared to non‐COPD non‐senescent cells (Figure S3). Upon exposure to BCL_XL_‐PROTAC for 72 h, senescent non‐COPD SAEC showed no change in BCL_2_ expression but displayed a significant reduction in BCL_XL_ expression at a concentration of 0.3 μM (Figure S4a). We then analysed the expression of senescence markers in senescent non‐COPD SAEC after treatment with BCL_XL_‐PROTAC. Both p21^CIP1^ and p16^INK4a^ expression and PAI‐1 secretion were significantly decreased in cells treated with 1 and 3 μM of BCL_XL_‐PROTAC (Figure S4b). The percentage of cells positive for β‐galactosidase fell from 26.8% ± 2.5% in untreated cells to 9.5% ± 4.1% in cells treated with 3 μM of BCL_XL_‐PROTAC, further confirming the reduction of senescent markers in senescent non‐COPD SAEC in response to treatment (Figure S4c). Next, we confirmed that the reduction of senescence markers after 72 h of treatment was associated with the activation of caspase 3/7 after 48 h of treatment, as shown by flow cytometry and fluorescence microscopy (Figures S4d,e and S5). To further confirm that the effect of BCL_XL_‐PROTAC is specific to senescent cells and safe for use on healthy cells, we treated aged‐matched non‐COPD non‐senescent SAEC with BCL_XL_‐PROTAC for 72 h and showed no reduction of BCL_2_ expression but a significant diminution of BCL_XL_ expression from 0.3 μM (Figure 3a). However, despite the degradation of BCL_XL_, healthy SAEC exhibited no significant changes in p21^CIP1^ and p16^INK4a^ expression, nor in PAI‐1 secretion (Figure 3b). The percentage of cells positive for β‐galactosidase was also unchanged after treatments (7.7% ± 3.2% in untreated cells vs. 5.6% ± 3.9% in cells treated with 3 μM of BCL_XL_‐PROTAC, Figure 3c). Additionally, analysis by flow cytometry and fluorescence microscopy indicated that the treatment of healthy SAEC with BCL_XL_‐PROTAC did not induce activation of caspase 3/7 (Figure 3d,e). In conclusion, BCL_XL_‐PROTAC is a potent senolytic agent that specifically eliminates senescent cells within a mixed population of senescent cells such as COPD SAEC, without causing any apparent detrimental effect on non‐COPD non‐senescent SAEC.

### 2.4 BCL_XL_ ‐PROTAC Suppressed Expression of Senescence Markers by Inducing Apoptosis of COPD Small Airway Fibroblasts

**FIGURE 4:**
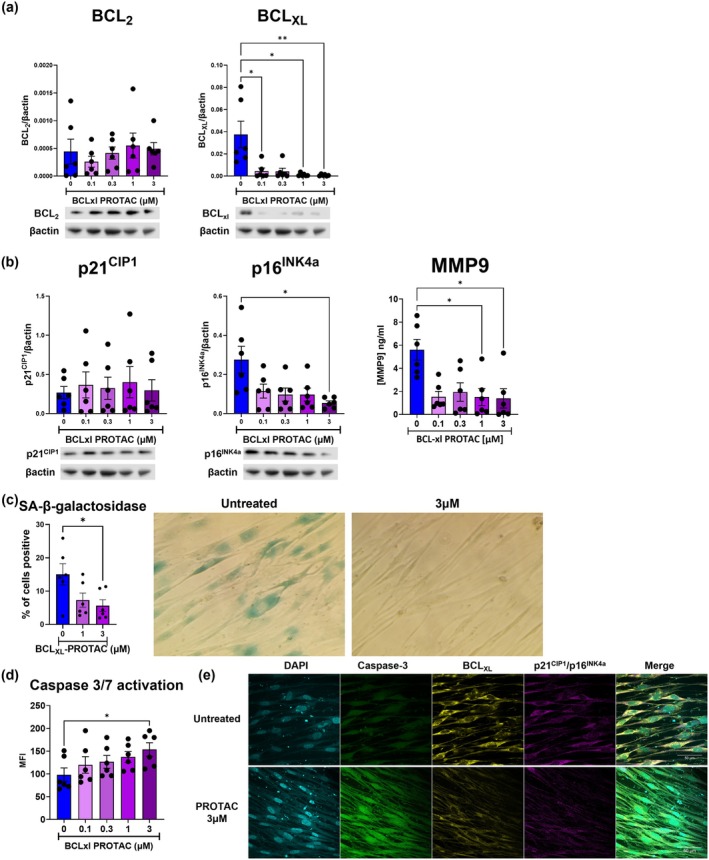
Degradation of BCL_XL_ by BCL_XL_‐PROTAC leads to the decreased expression of senescence markers in COPD SAF. SAF derived from COPD lungs were treated with 0.1 μM–3 μM of BCL_XL_‐PROTAC for 24 to72 h. (a) After 72 h, the expression of BCL_2_ and BCL_XL_ was measured in cell lysates by Western Blot. (b) The expression of p21^CIP1^ and p16^INK4a^ was measured by Western Blot and the production of the SASP MMP‐9 was measured in cell media by ELISA. (c) After 72 h, COPD SAF were fixed and stained for senescence associated β‐galactosidase and imaged by light microscopy. (d) After 48 h, the activity of caspase 3/7 was quantified by flow cytometry. (e) After 48 h, cells were fixed and stained for DAPI (turquoise), caspase 3/7 (green), BCL_XL_ (yellow) and p21^CIP1^/p16^INK4a^ (pink) and imaged by fluorescent microscopy. Images are representative of five different experiments conducted on five different patients. Representative Western blots of (a) BCL_XL_ and BCL_2_ and (b) p21^CIP1^ and p16^INK4a^ expression are shown, quantified, and normalised to β‐Actin. Panels (a) and (b) are derived from the same Western blot using the same samples, treatments and timepoint. Data are expressed as mean ± SEM, analysed by Kruskal–Wallis test. *p < 0.05. **p < 0.01.

Cellular senescence is a complex cellular process that can depend on the specific trigger or cell type. Consequently, the effect of senolytic therapies can vary in different cell types (Walters 2024). In our study, we demonstrated that BCL_XL_‐PROTAC is a potent senolytic on COPD and senescent SAEC, therefore we aimed to analyse whether this compound exerts a similar effect on COPD small airway fibroblasts (SAF) as these cells are also considered to be important in the development of small airway fibrosis in COPD and have been shown to have a senescent and pro‐fibrotic phenotype (Wrench et al. 2024). Therefore, we cultured COPD SAF with increasing concentrations of BCL_XL_‐PROTAC for up to 72 h and observed that while BCL_2_ expression remained unchanged, again, there was a significant decrease in BCL_XL_ expression in a concentration‐dependent manner (Figure 4a). Next, we analysed whether the degradation of BCL_XL_ in COPD SAF would lead to changes in the expression of senescence markers. We found that the expression of p16^INK4a^ significantly decreased in cells treated with 3 μM of BCL_XL_‐PROTAC, whereas p21^CIP1^ levels did not change (Figure 4b). Although we did not detect any change in PAI‐1 secretion in COPD SAF (data not shown), it is worth noting that PAI‐1 is primarily produced in the lungs of COPD patients by epithelial cells and alveolar type 2 cells and not fibroblasts (Adnot et al. 2020). Therefore, we measured the production of another SASP factor known to be produced by SAF: matrix metalloproteinase (MMP)‐9 (Wang et al. 2024). We found that MMP‐9 secretion was reduced in cell media of cells treated with 1 and 3 μM of BCL_XL_‐PROTAC (5.6 ± 0.9 ng/mL in untreated cells vs. 1.4 ± 0.8 ng/mL in cells treated with 3 μM). Additionally, we assessed the percentage of SAF positive for SA‐β‐galactosidase which decreased from 15.1% ± 3.2% in untreated cells to 5.6% ± 1.8% in cells treated with 3 μM of BCL_XL_‐PROTAC (Figure 4c). Furthermore, we analysed the activation of caspase 3/7 after 48 h of treatment and confirmed that COPD SAF exhibit increased activation of the caspase 3/7 when treated with 3 μM of BCL_XL_‐PROTAC, compared to untreated cells (MFI = 98.1 ± 15.0 in untreated cells vs. MFI = 153.7 ± 14.89 in cells treated with BCL_XL_‐PROTAC, Figure 4d,e). In summary, these results confirm that BCL_XL_‐PROTAC exerts its senolytic activity through the degradation of BCL_XL_ in both SAEC and SAF.

### 2.5 Clearance of Senescent Cells by BCL_XL_ ‐PROTAC Induced Cell Proliferation of COPD SAEC and SAF

**FIGURE 5:**
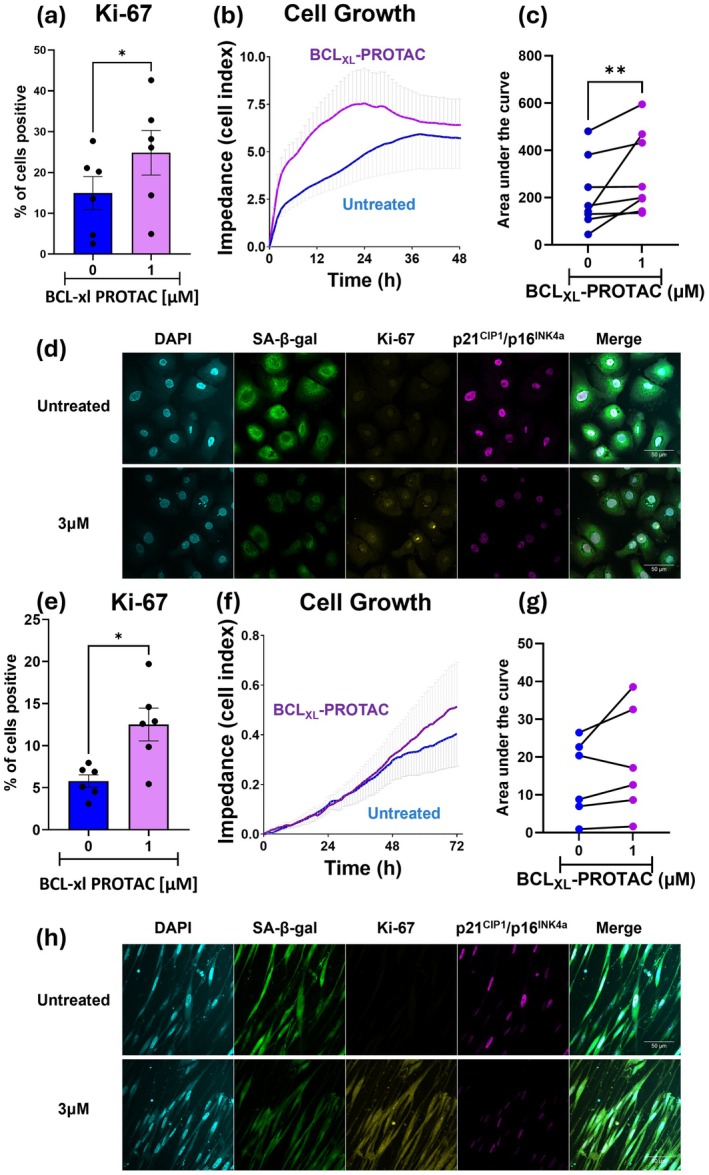
Degradation of BCL_XL_ by BCL_XL_‐PROTAC leads to the increased proliferation of COPD SAEC and fibroblasts. SAEC and SAF derived from COPD lungs were treated with 1 μM or 3 μM of BCL_XL_‐PROTAC for up to 72 h. After 72 h, the expression of Ki‐67 was measured in (a) SAEC and (e) SAF by flow cytometry. Proliferation rate of (b) SAEC and (f) SAF were measured by x‐Celligence technology. The area under the curve was measured in (c) SAEC and (g) SAF. After 72 h, (d) SAEC and (h) SAF were fixed and stained for DAPI (turquoise), SA‐β‐galactosidase by CellEvent (green), Ki‐67 (yellow) and p21^CIP1^/p16^INK4a^ (pink) and imaged by fluorescent microscopy. Images are representative of 5 experiments. Data are expressed as mean ± SEM, analysed by Kruskal–Wallis or Wilcoxon test as appropriate. **p < 0.01.

One aim of senolytic drugs is to eliminate senescent cells, thereby creating space for healthy cells to proliferate which would facilitate tissue regeneration and repair (von Kobbe 2019). To determine whether the clearance of senescent COPD SAEC and SAF by BCL_XL_‐PROTAC stimulates cell regeneration, both types of cells were treated with BCL_XL_‐PROTAC. Their proliferation status was assessed by measuring the proportion of cells expressing Ki‐67 and by quantifying cell growth and adhesion through electrical impedance using X‐Celligence (Figure 5). COPD SAEC treated with 3 μM of BCL_XL_‐PROTAC for 72 h exhibited a significantly higher proportion of Ki‐67 positive cells compared to untreated cells (21.4% ± 2.4% in untreated cells vs. 40.4% ± 2.3% in cells treated with BCL_XL_‐PROTAC). Cells were then treated with BCL_XL_‐PROTAC for 48 h and loaded in the X‐Celligence. We observed that COPD SAEC treated with BCL_XL_‐PROTAC showed increased cell growth compared to non‐treated cells, with a maximum effect after 24 h where the area under the curve showed a significant effect of BCL_XL_‐PROTAC on COPD SAEC growth (Figure 5b,c). Additionally, fluorescence microscopy confirmed that COPD SAEC treated with 3 μM of BCL_XL_‐PROTAC exhibited decreased expression of SA‐β‐galactosidase and p21^CIP1^/p16^INK4a^ along with an increased expression of Ki‐67 (Figure 5d). Consistent with the results obtained on COPD SAEC, COPD SAF treated with BCL_XL_‐PROTAC for 72 h also showed an increased proportion of Ki‐67 positive cells compared to untreated cells (5.8% ± 0.78% of untreated cells vs. 20.28% ± 5.0% in cells treated with BCL_XL_‐PROTAC) (Figure 5e). COPD SAF cultured with 3 μM of BCL_XL_‐PROTAC also showed increased cell growth compared to non‐treated cells, with the maximum effect observed after 24 h (Figure 5f,g). Fluorescent microscopy further confirmed that COPD SAF treated for 72 h with BCL_XL_‐PROTAC exhibited decreased expression of SA‐β‐galactosidase and p21^CIP1^/p16^INK4a^, along with an increased expression of Ki‐67 (Figure 5h). Overall, these results confirm that BCL_XL_‐PROTAC, as a senolytic agent, induces apoptosis of senescent cells which may stimulate the proliferation of healthy cells, potentially favouring lung regeneration and repair.

### 2.6 Validation of the Effect of BCL_XL_ ‐PROTAC on Precision‐Cut Lung Slices

**FIGURE 6:**
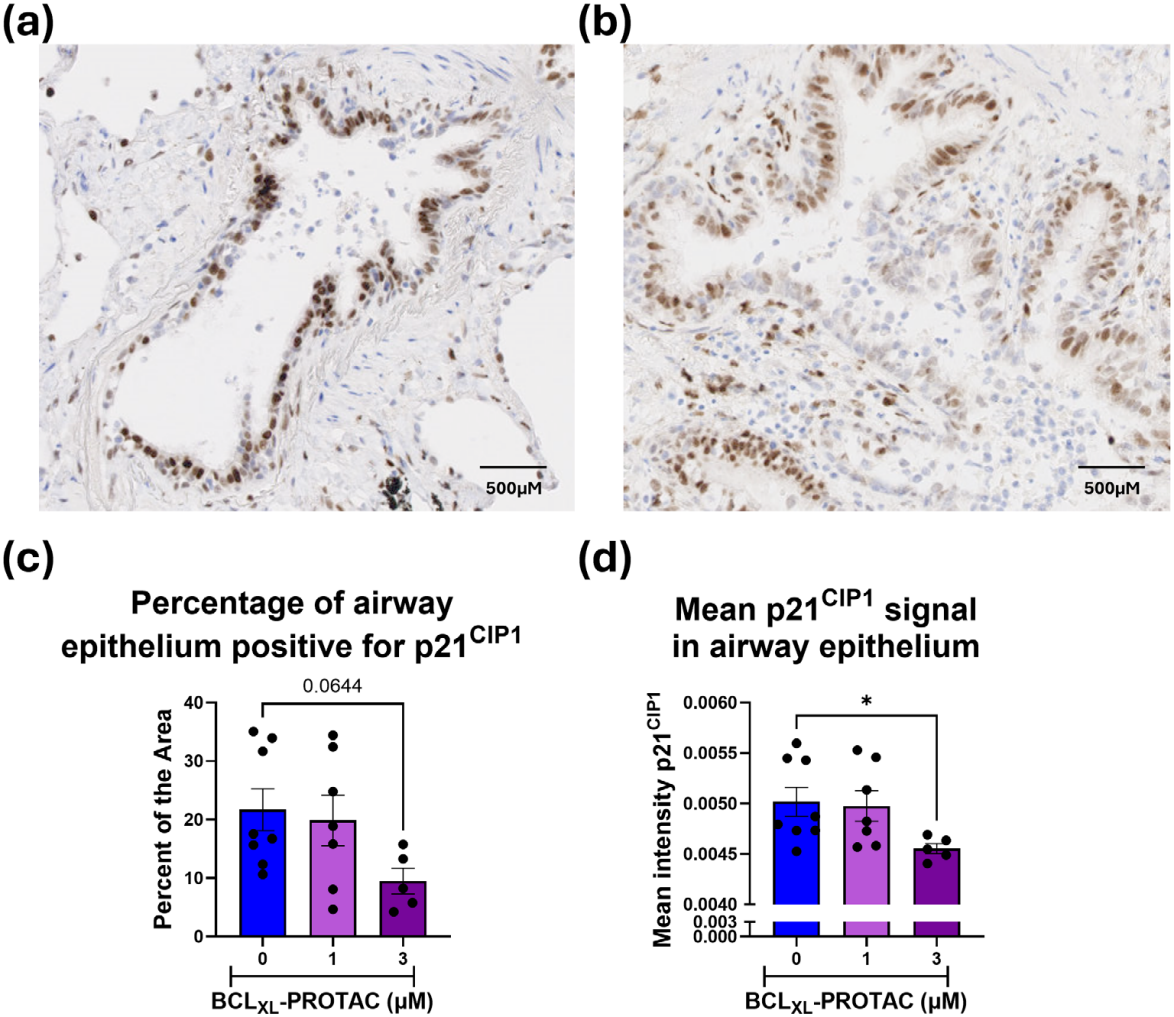
Degradation of BCL_XL_ by BCL_XL_‐PROTAC leads to the decreased expression of senescent markers in COPD PCLS. PCLS derived from COPD were treated with 1 or 3 μM of BCL_XL_‐PROTAC for 48 h. PCLS were fixed and slices in 3 μm sections. PCLS were then stained for H&E and p21^CIP1a^ and imaged using the NanoZoomer 2.0HT. Small airways were located on (a) untreated and (b) treated PCLS and p21^CIP1^ signal was analysed using Image J. (c) The percentage of the epithelium area positive for p21^CIP1^ and (d) the mean intensity of p21^CIP1^ signal in the epithelium was analysed. Images are representative of 5 experiments. Data are expressed as mean ± SEM, analysed by Kruskal–Wallis. *p < 0.05.

To validate the results obtained in vitro in cells derived from COPD lung tissue, a more complex model of lung tissue was necessary. PCLS provide an ex vivo model that preserves lung architecture, resident cell types, signalling, and responses, thus providing a functional heterogenous environment (Sompel et al. 2023). In this study, we generated PCLS from COPD lung tissue and cultured these with BCL_XL_‐PROTAC. We assessed the effect of BCL_XL_‐PROTAC by staining and quantifying p21^CIP1^ using immunohistochemistry and qRT‐PCR (Figure 6). In untreated COPD PCLS, the staining of p21^CIP1^ was primarily localised within the small airway epithelium (Figure 6a). These findings confirm that cellular senescence is a highly heterogenous phenomenon affecting various regions of the lung rather than presenting uniformly throughout the entire organ. Treatment of COPD PCLS with BCL_XL_‐PROTAC did not modify the overall expression of p21^CIP1^; however, this is likely due to the cell‐specific effect of BCL_XL_‐PROTAC (Figure S6). Thus, we analysed the percentage of the airway epithelium positive for p21^CIP1^ and its mean intensity, comparing the untreated PCLS to those treated with BCL_XL_‐PROTAC. Overall, treatment with 3 μM of BCL_XL_‐PROTAC resulted in a decrease of the p21^CIP1^ signal localised in the airway epithelium (Figure 6b). The proportion of the area of the airway epithelium positive for p21^CIP1^ decreased from 21.7% ± 3.5% in untreated PCLS to 9.47% ± 2.2% in PCLS treated with BCL_XL_‐PROTAC (Figure 6c) indicating a reduction in the number of epithelial cells positive for p21^CIP1^. Moreover, the mean intensity of p21^CIP1^ staining significantly decreased in PCLS treated with 3 μM BCL_XL_‐PROTAC, confirming a reduction in the overall p21^CIP1^ signal in response to the treatment (Figure 6d). Thus, the results support the therapeutic potential of BCL_XL_‐PROTAC as a senolytic agent that specifically targets senescent cells in vitro and in PCLS, leading to a decrease in the number of senescent cells, particularly around the airway epithelium.

## 3 Discussion

The selective clearance of senescent cells holds promise for the treatment of age‐related diseases. The accumulation of senescent cells in tissues and organs is associated with the development and severity of age‐related diseases, including COPD. Targeting BCL_XL_ has shown significant effects in vitro and in mouse models; however, concerns have been raised regarding dose‐limiting thrombocytopenia associated with treatments such as navitoclax (Chang et al. 2016; de Vos et al. 2021; Tarantini et al. 2021). PROTAC technology combined with BCL_XL_ targeting aims to reduce the overall drug exposure and minimise the risk of side effects. It has demonstrated effectiveness in targeting BCL_XL_ in vitro for cancer treatment, but its impact on age‐related diseases has yet to be explored. In this study, we successfully used a BCL_XL_‐PROTAC that targets BCL_XL_ to CRBN E3 ligase, which is not expressed in platelets, on senescent COPD SAEC and SAF. We demonstrated that BCL_XL_‐PROTAC reduced senescent markers in vitro in small airway cells and in PCLS derived from COPD patients. This suggests his efficacy and safety to use therapeutically. Additionally, we observed that cells surviving treatment with BCL_XL_‐PROTAC showed increased proliferation, pointing to the potential for promotion of lung regeneration.

One of the hallmarks of ageing is the accumulation of senescent cells, which are notably resistant to apoptosis due to the upregulation of BCL_2_ anti‐apoptotic family (Yosef et al. 2016). This family includes BCL_2_, BCL_XL_, BCL_W_, MCL‐1 and BFL‐1/A1, all of which regulate apoptosis by binding to the pro‐apoptotic BCL proteins, BAK and BAX, located at the mitochondrial outer membrane (Kale et al. 2018). Dysregulation of the balance between pro‐ and anti‐apoptotic BCL proteins has been observed in lung tissues of COPD patients (Siganaki et al. 2010). Targeting the BCL_2_ antiapoptotic family of proteins presents significant potential for COPD. In a mouse model of emphysema, the administration of navitoclax prevented the accumulation of senescent cells within the lungs and mitigated the decline in lung function and degradation of alveolar morphology (Mikawa et al. 2018). Our results show that small airway epithelial cells and fibroblasts from COPD patients treated with BCL_XL_‐PROTAC showed increased activation of caspase 3/7, leading to increased apoptosis, and decreased expression of senescence markers. This suggests that treating COPD patients with BCL_XL_‐PROTAC could have a beneficial effect on lung cellular senescence, without the harmful side effects associated with navitoclax. The PROTAC technology was developed precisely to minimise the high risk of thrombocytopenia seen with other treatments. Although we did not evaluate the impact of BCL_XL_‐PROTAC on platelets in this study, a Phase I trial involving cancer patients indicated that the risk of grade 4 thrombocytopenia was very limited (Mahadevan et al. 2025). Given that ageing is linked to an increased risk of developing thrombocytopenia, the safety of BCL_XL_‐PROTAC as a treatment should be further investigated in older populations with or without platelets deficiency. Although our findings did not show an effect of BCL_XL_‐PROTAC on BCL_2_ expression, we cannot rule out its impact on other members of the BCL_2_ family proteins. For example, a degrader of BCL_XL_, DT2216, reduced expression of BCL_XL_ in MOLT‐4 cells at low concentrations (~80 nM) but expression of cleaved caspase 3 was upregulated only at 100 and 300 nM (Zhang et al. 2020). Although the study did not analyse senescence markers, it reflected a similar pattern to our observations regarding the degradation of BCL_XL_ and its effects on inducing apoptosis and reducing senescence markers. Additionally, in an in vitro model of senescence induced in epithelial cells or fibroblasts by irradiation, oncogenes or drugs, a subset of cells overexpressing MCL‐1 was resistant to treatment with classical drugs targeting BCL_2_, BCL_XL_ or BCL_W_, including navitoclax (Rysanek et al. 2022). In COPD patients, the expression and function of the different members of the BCL_2_ family have yet to be fully characterised, which would help elucidate the efficacy of BCL_XL_‐PROTAC in our model. Furthermore, this suggests that combining various senolytics could be beneficial in completely eliminating senescent cells while allowing the use of lower, and potentially less toxic, drug doses, thereby enhancing the treatment potential for COPD patients.

Most studies examining the effects of targeting BCL_XL_ have been conducted using commercially available cancer cell lines. In this study, we used small airway epithelial cells and small airway fibroblasts isolated from lung resections from either healthy donors or COPD patients. However, one limitation of our study is that we analysed these cells in vitro as monolayers instead of using air‐liquid interface (ALI) culture. ALI system better reflects the complex cellular composition of the respiratory tract and closely mimic physiological conditions. Furthermore, the ALI model can be constructed from patient‐derived cells to model pathophysiology conditions (Baldassi et al. 2021). It has been shown that COPD bronchial epithelial cells in an ALI system maintain the senescent phenotype observed in monolayer culture, characterised by the elevated expression of p16^INK4a^ and production of SASP factors like CXCL8 and CCL5. Treatment of these cells with the senolytic combination of dasatinib and quercetin leads to a decreased expression of p16^INK4a^ associated with improved epithelial barrier integrity and reduced expression of SASP (Baker et al. 2025). To maintain consistency across different culture conditions and parameters in our analysis, including X‐Celligence,which can only be measured in a monolayer system, we studied the effect of BCL_XL_‐PROTAC in a monolayer model. To address the limitation of the monolayer approach, we employed precision cut lung slices (PCLS) that replicate the architecture of the lung tissue, including the small airways, the blood vessels and parenchyma allowing us to study resident cell types in the context of the whole tissue (Koziol‐White et al. 2024). PCLS enable the analysis of tissue remodelling and small airways mechanics in COPD. Notably, PCLS derived from COPD patients demonstrated small airway hyperresponsiveness due to reduce stiffness of the airway wall and diminished parenchymal retraction forces (Maarsingh et al. 2019). In a mouse model of lung fibrosis, PCLS have been used to screen and identify potential senolytic drugs that eliminate p16^INK4a+^ fibroblasts (Lee et al. 2024). To our knowledge, we are the first to demonstrate that the use of a senolytic, specifically BCL_XL_‐PROTAC, leads to decreased expression of senescence markers in PCLS derived from COPD patients, particularly localised in the small airway epithelium. These findings align with our in vitro results and confirm the capacity of BCL_XL_‐PROTAC to target senescent cells within a complex tissue environment, supporting its translational and therapeutical potential.

In addition to its impact on small airway epithelial cells, BCL_XL_‐PROTAC has shown potent senolytic activity against small airway fibroblasts in COPD. A subset of small airway fibroblasts exhibits a senescence phenotype and is believed to contribute to small airway diseases and fibrosis in COPD. These senescent fibroblasts express genes associated with oxidative stress response, fibrosis and mitochondrial dysfunction (Wrench et al. 2024). Furthermore, the transplantation of senescent human fibroblasts into the lungs of immunodeficient mice induces progressive lung fibrosis, which correlates with an increased number of senescent mouse fibroblasts within the lungs. In contrast, the transplantation of non‐senescent fibroblasts had no effect (Hernandez‐Gonzalez et al. 2023). Senolytic drugs have been shown to limit the development of fibrosis, particularly in models of idiopathic pulmonary fibrosis (IPF). In a mouse model of lung fibrosis induced by bleomycin, the elimination of BCL_2_^+^ cells using navitoclax induced apoptosis of fibrotic fibroblasts and reversed lung fibrosis, significantly reducing collagen deposition in the lung. In a mouse model of fibrosis, alveolar epithelial cells exhibit increased expression of senescence markers and secrete an increased level of SASP. Treatment with dasatinib and quercetin, both in and ex vivo, leads to decreased expression of senescence markers in alveolar epithelial cells and mitigates the development of lung fibrosis (Lehmann et al. 2017). Our results indicate that treating COPD small airway fibroblasts with BCL_XL_‐PROTAC decreases the expression of the senescence marker p16^INK4a^ and reduces the production of the SASP factor MMP9. Although we did not measure the effect of BCL_XL_‐PROTAC on fibrosis in vitro or in PCLS, these findings confirm the effect of BCL_XL_‐PROTAC across various lung cell types. Future research should focus on the impact of this BCL_XL_‐PROTAC on lung fibrosis in COPD. Further studies are needed to evaluate the efficacy and safety of BCL_XL_‐PROTAC in the context of emphysema. Studies indicate that short telomeres are a genetic factor that can increase patients' susceptibility to developing emphysema (Alder et al. 2011). Therefore, the effects of BCL_XL_‐PROTAC in this patient population will require further characterisation, as eliminating these cells may not lead to cell regeneration and could potentially result in increased fibrosis.

Our results indicate that treatment with BCL_XL_‐PROTAC enhances the proliferation rate of small airway epithelial cells and small airway fibroblasts derived from COPD lungs. The use of senolytics to clear senescent cells is believed to create a more favourable environment with reduced inflammation, allowing non‐senescent cells to proliferate without directly affecting their function (Atlante et al. 2025). Our results indicate that BCL_XL_‐PROTAC induces the apoptosis of COPD cells, resulting in a reduction of senescence marker expression. Additionally, we demonstrated that treating COPD cells with BCL_XL_‐PROTAC leads to an upregulation of proliferation markers. To further explore the potential effect of BCL_XL_‐PROTAC on cell proliferation and lung rejuvenation, additional studies in both mouse and human models are required. Particularly, the impact of BCL_XL_‐PROTAC on lung stem cells needs to be characterised. In a mouse model of lung fibrosis and in three‐dimensional human lung tissue culture, the combination treatment with dasatinib and quercetin resulted in the apoptosis of fibrotic AT2 cells, which was associated with reduced fibrosis markers and inflammation, alongside an increase in alveolar epithelial markers (Lehmann et al. 2017). Currently, there are no studies that have analysed the effect of senolytics on the proliferation and differentiation of lung stem cells, which is essential for understanding whether and how senolytics may contribute to lung rejuvenation.

In conclusion, our data demonstrated the potency of the novel senolytic, BCL_XL_‐PROTAC, in clearing small airway senescent cells in the age‐related disease COPD. We showed that BCL_XL_‐PROTAC specifically targets BCL_XL_, rather than BCL_2_, leading to the apoptosis of senescent small airway epithelial cells and fibroblasts. This process results in a reduction of the expression of senescent markers, including p21^CIP1^ and p16^INK4a^, as well as the secretion of proinflammatory factors and the increased expression of proliferation markers. The accumulation of senescent cells is a hallmark of ageing and is a significant pathophysiological mechanism. Our results show that targeting BCL_XL_ can reduce disease progression, limit pulmonary inflammation, and may contribute to lung rejuvenation. Overall, we have highlighted that BCL_XL_‐PROTAC holds great promise for treating COPD and potentially other age‐related diseases.

## 4 Methods

### 4.1 Reagents and Antibodies

Antibodies against the following were used for immunoblotting: βactin (Santa Cruz Biotechnology, Santa Cruz, CA), BCL_XL_ (54H6, MAB2764), BCL_2_ (124, MAB15071), p21^CIP1^ (12D1, MAB2947), all from Cell Signaling Biotechnology (Beverly MA), and p16^INK4a^ (EPR24167‐43, ab270058) from AbCam. Anti‐rabbit and anti‐mouse secondary antibodies were purchased from Cell Signaling Biotechnology (Beverly, MA). Caspase 3/7 activation was measured by flow cytometry using the CellEvent Caspase‐3/7 Detection Reagents (Invitrogen, MA, USA). Pacific Blue anti‐human Ki‐67 was used for flow cytometry from Biolegend (CA, USA). BCL_XL_ and BCL_2_ degradation were measured using AlphaLISA *SureFire Ultra* Human and Mouse Total BCL_XL_ Detection Kit (Revvity, ALSU‐TBCLXL‐A‐HV) and AlphaLISA *SureFire Ultra* Human Total BCL_2_ Detection Kit (Revvity, ALSU‐TBCL2‐A‐HV) (Revvity, MA, USA). Proteasome inhibitor (MG132) was purchased from Sigma Aldrich, (Germany, 474790‐1MG). We have used the PZ15227, called BCL_XL_‐PROTAC throughout the manuscript, which targets BCL_XL_ to the cereblon (CRBN) E3 ligase for degradation (He et al. 2020).

### 4.2 Cell Culture

Cells were extracted from lung tissue from subjects undergoing lung resection surgery at the Royal Brompton Hospital and Hammersmith Hospital, London. The subjects were matched for age (Table 1). All subjects gave informed written consent, and the study was approved by the London‐Chelsea Research Ethics committee (study 15/SC/0101 and 17/WA/0161). Primary small airway epithelial cells (SAEC) were cultured as monolayers in Small Airway Epithelial Cell Growth Medium (Promocell, Germany) on collagen (1% w/v) coated plates (Devulder et al. 2025). Primary small airway fibroblasts were cultured as monolayer in Dulbecco's Modified Eagle Medium (DMEM) complemented with 10% (v/v) Foetal Bovine Serum, 1% (v/v) of L‐glutamin, 1% (v/v) penicillin–streptomycin and 1% (v/v) amphotericin (Wrench et al. 2024).

**TABLE 1:** Characteristics of study subjects.

Characteristics	Non‐COPD SAEC (*n* = 6)	COPD SAEC (*n* = 6)	COPD SAF (*n* = 6)	PCLS (*n* = 8)
Age (years)	64.3 ± 8.9	68.3 ± 4.5	64.5 ± 3.4	67.13 ± 3.56
Sex (M:F)	03:03	05:01	06:00	02:06
FEV_1_ (L)	2.28 ± 0.38	1.275 ± 0.23*	1.263 ± 0.30*	1.30 ± 0.25
FEV_1_ (% predicted)	101.3 ± 6.2	57.38 ± 11.02**	35.63 ± 8.39****	55.96 ± 9.94
FVC (L)	3.2 ± 0.44	3.48 ± 0.23	3.35 ± 0.39	2.75 ± 0.20
FEV_1_:FVC	0.73 ± 0.016	0.35 ± 0.05***	0.38 ± 0.07***	0.46 ± 0.07
Pack‐years^a^	0	40.67 ± 7.15	58.17 ± 11.9	28.38 ± 5.59

*Note:* Patients with COPD are all ex‐smoker and were categorised according to Global Initiative for Chronic Obstructive Lung Diseases. Data are expressed as mean ± SEM, **p > 0.01. ***p > 0.001.

Abbreviations: F, female; FEV_1_, forced expiratory volume in 1 s; FVC, forced vital capacity; M, male.

^a^ Number of cigarettes smoked per day/20 × duration of smoking.

### 4.3 Assessment of BCL_XL_ Degradation by BCL_XL_ ‐PROTAC

SAEC were plated into 96‐well plates and kept for 24 h to adhere and reach 80% confluency. Cells were treated for 24 h with increasing concentrations of BCL_XL_‐PROTAC as indicated. For experiments with the proteasome inhibitor MG132 or cereblon binder (CRBN), cells were co‐treated with BCL_XL_‐PROTAC and MG132 (3 μM) or BCL_XL_‐PROTAC and CRBN (10 μM), and incubated for 24 h. Cells were lysed, and degradation was measured by AlphaLisa according to the manufacturer's protocol. Briefly, cells were lysed in Lysis Buffer for 10 min on an orbital shaker. Acceptor Beads were added to the lysates followed by 1 h incubation at room temperature. Next, Donor Beads were added and followed by 1 h incubation at room temperature in the dark. The plates were read on EnVision Multimode plate reader at 615 nm (Perkin Elmer). Percentage of degradation was calculated using the equation: ((max absorbance−sample absorbance)/(max absorbance−min absorbance)) * 100.

### 4.4 Induction of Replicative Senescence

Replicative senescence was induced in SAEC isolated from non‐COPD donors. Cells were seeded at 75% confluence on collagen‐coated plates in small airway epithelial cell growth medium. Cells were fed twice a week, and the media were changed weekly. After 14 days in culture, cells were > 95% positive for senescence associated (SA)‐β‐galactosidase, produced high levels of PAI‐1 and expressed increased levels of p21^CIP1^ and p16^INK4a^ (Figure S2).

### 4.5 Caspase Activation Assay

Cells were cultured in 24‐wells plates in 500 μL of complete cell medium per well (50,000 cells per well). Cells were exposed to 0.1 μM to 3 μM of BCL_XL_‐PROTAC for 24 to 72 h. After treatment, cells were trypsinised and stained with the CellEvent Caspase‐3/7 Detection Reagents for 30 min and cell viability was assessed with SYTOX AADvanced Dead Cell Stain Kit (Invitrogen, MA, USA). Cells were analysed using the Canto II flow cytometer (Beckton Dickinson Biosciences, Franklin Lakes, NJ) and results were expressed as mean fluorescence intensity of the CellEvent using FlowJo (Beckton Dickinson Biosciences, Franklin Lakes, NJ) (Figure S7a).

### 4.6 Proliferation Assays

Cell proliferation was assessed by measuring the expression of a marker of proliferation, Ki‐67, and by X‐Celligence (Agilent Technologies, CA, USA). For Ki‐67, cells were seeded and exposed to increasing concentrations of BCL_XL_‐PROTAC for 24 to 72 h. Cells were then trypsinised and fixed using 70% (v/v) ethanol before staining with Pacific Blue anti‐human Ki‐67 (Biolegend, CA, USA). The percentage of cells positive for Ki‐67 was then analysed with the Canto II flow cytometer and FlowJo (Beckton Dickinson Biosciences, Franklin Lakes, NJ) (Figure S7b).

For X‐Celligence assay, cells were first plated on collagen‐plated wells and stimulated for 48 h with 1 μM of BCL_XL_‐PROTAC. Cells were then trypsinised and seeded on 96‐wells E‐plates coated with collagen (1% w/v) at a density of 10,000 cells/well. The plates were then loaded on the x‐Celligence instrument and the changes in the impedance of the plates were monitored over 72 h. Results were then converted to a Cell Index that reflects the rate of proliferation of the cells.

### 4.7 Western Blot Analysis

250,000 cells were seeded in 6‐wells plates and exposed to 0.1 μM to 3 μM of BCL_XL_‐PROTAC for 72 h. Protein extracts were prepared using RIPA buffer (Sigma: 150 nM NaCl, 1.0% IGEPAL CA‐630, 0.5% (w/v) sodium deoxycholate, 0.1% (w/v) SDS, and 50 mM Tris, pH 8.0) containing antiproteases (Roche, Welwyn Garden City, UK). Protein concentrations were determined using Bio‐Rad Protein Assay Dye Reagent (BioRad, CA, USA). Samples were loaded onto NuPAGE 4%–12% (w/v) Bis‐Tris Protein Gels (Invitrogen) and run with NuPAGE MOPS SDS Running (Invitrogen) according to the NuPAGE Novex electrophoresis program. Proteins were transferred to Nitrocellulose Blotting Membranes (Invitrogen) and incubated overnight at 4°C with the primary antibody in blocking buffer. Next day, the membranes incubated with anti‐mouse or anti‐rabbit secondary antibody conjugated with horseradish peroxidase (HRP) and analysed by chemiluminescence (ECL Plus; GE Healthcare, Hatfield, UK) using an Odyssey CLx scanner and the ImageStudio Software (LI‐COR Biosciences). Protein signals were normalised to β‐actin signal.

### 4.8 SA‐β‐Galactosidase Staining

SAEC or SAF were plated into 24‐well plates (50,000 cells per well) and left for 24 h to adhere. Cells were treated with increasing concentrations of BCL_XL_‐PROTAC for 72 h and the activity of SA‐β‐galactosidase was determined according to the manufacturer's instructions (ab65351; Abcam). Briefly, cells were fixed and stained for β‐galactosidase with its substrate X‐galactosidase for 24 h at 37°C. Cells were imaged by light microscopy and the percentage of cells positive for β‐galactosidase (stained in blue) was calculated.

### 4.9 ELISA

SAEC or SAF were plated and treated with BCL_XL_‐PROTAC for 72 h. Cell media were recovered and concentration of PAI‐1 or MMP‐9 were measured by ELISA using kits from R&D systems (Minneapolis, MN, USA). Concentrations of PAI‐1 and MMP‐9 were measured according to manufacturer's instructions and calculated using the standard curve.

### 4.10 Fluorescent Microscopy

SAEC and SAF were seeded on coverslips coated with 1% (w/v) collagen and treated for 48 or 72 h with 1 or 3 μM of BCL_XL_‐PROTAC (50,000 cells per well). Cells were fixed and stained with two mixes: mix 1 consisting of CellEvent Caspase‐3/7 Detection Reagents (Absorption max 502/530 nm, Invitrogen, MA, USA), BCL_XL_ (54H6, MAB2764, Cell Signalling Biotechnology Beverly, MA), p21^CIP1^/p16^INK4a^ (WA‐1, MA1‐33926 / 4A10E1, MA5‐38494, Invitrogen, MA, USA), or mix 2 consisting of CellEvent Senescence Green Flow Cytometry Assay Kit (Absorption max 490/514 nm, Invitrogen, MA, USA), Ki‐67 (SP6, MA5‐14520, Invitrogen, MA, USA), p21^CIP1^/p16^INK4a^ (WA‐1, MA1‐33926 / 4A10E1, MA5‐38494, Invitrogen, MA, USA). Cells were incubated with DAPI (Abcam, Cambridge, United Kingdom) and secondary antibodies AF568 Goat anti‐Rabbit IgG and AF647 Goat anti‐Mouse IgG1 (both from Invitrogen, MA, USA). Cells were then imaged by confocal microscopy using the Leica Stellaris 5 (Leica, Wetzlar, Germany) and analysed by ImageJ software.

### 4.11 Generation and Stimulation of Precision‐Cut Lung Slices (PCLS)

Lung tissues were kept on ice and inflated with a 3% agarose (w/v) solution in HBSS. 8 mm diameter cores were made and loaded onto a Krumdiek Tissue microtome slicer (Alabama Research and Development) in HBSS buffer (Brown et al. 2013). Slices of 300 μm were recovered in 24‐wells plate and incubated overnight in RPMI supplemented with 1% (v/v) of L‐glutamine, 1% (v/v) penicillin–streptomycin and amphotericin, 0.1% Gentamycin. Tissue slices were then cultured with 1 or 3 μM of BCL_XL_‐PROTAC for 48 h. After treatment, PCLS were fixed in neutral buffered formalin and stored in 70% (v/v) ethanol before being embedded in paraffin. PCLS sections of 3 μm were made using a microtome (ThermoFisher Scientific, Waltham, MA). Haematoxylin and Eosin (H&E) staining was performed on Leica H&E autostainer. The sections were deparaffinised, hydrated and then heat mediated antigen retrieval was performed in EDTA based pH 9.0 solution. The endogenous peroxidase was quenched with 3% (v/v) hydrogen peroxide. The sections were incubated with rabbit monoclonal to p21^Waf1/Cip1^ (from Cell Signaling Biotechnology Beverly, MA) and subsequently incubated with anti‐rabbit IgG conjugated with polymeric horseradish peroxidase linker (Leica Bond Polymer Refine Detection, DS9800). 3,3′‐diaminobenzidine (DAB) was used as the chromogen and the sections were then counterstained with haematoxylin and mounted with DPX. Immunohistochemistry (IHC) staining was performed on Leica BOND RX. The stained‐glass slides were scanned with NanoZoomer 2.0HT (Hamamatsu, Japan). NDP.scan 3.4.1 software was used for digital image acquisition and NDP.view2 software was used for image viewing. Images were analysed using Image J.

### 4.12 Statistical Analysis

Data are expressed as mean ± SEM. Results were analysed using Two‐Way ANOVA, Mann–Whitney, Wilcoxon or Kruskal–Wallis tests as appropriate. GraphPad Prism 9.2 software (GraphPad software, La Jolla, CA) was used for analysis. Values of *p* ≤ 0.05 were considered statistically significant.

## Author Contributions

P.J.B., L.E.D. and J.V.D. designed research; J.V.D., P.S.F., E.K. and H.K. performed research; J.V.D., P.S.F. and A.S. analysed the data; R.L., N.R., L.O., M.A.‐S. and P.V. contributed new reagents or analytic tools; J.V.D. wrote the paper.

## Funding

This work was supported by Medical Research Centre.

## Conflicts of Interest

Dr. Lina, Odqvist, Dr. Ewa Kolosionek, Dr. Raphael Lemaire, and Dr. Neetu Razdan are employed by AstraZeneca and may own company shares/stock options.

## Supporting information


**Figure S1:** Treatment with BCL_XL_‐PROTAC does not modify proinflammatory chemokines and cytokines production by COPD SAEC. SAEC derived from COPD lungs were treated with 0.1 μM to 3 μM of BCL_XL_‐PROTAC for 72 h. The production of CXCL8 (on the left) and IL‐6 (on the right) was measured in cell media by ELISA. Data are expressed as mean ± SEM (*n* = 6), analysed by Kruskal–Wallis test.
**Figure S2:** Treatment with BCL_XL_‐PROTAC leads to the activation of caspase 3/7 and decreased expression of BCL_XL_ in COPD SAEC. SAEC derived from COPD lungs were treated with 3 μM of BCL_XL_‐PROTAC for 48 h. Cells were fixed and stained for DAPI, caspase 3/7, BCL_XL_ and p21^CIP1^/p16^INK4a^ and imaged by fluorescent microscopy. The mean intensity of caspase 3/7 (on the left) and BCL_XL_ (on the right) was analysed and quantified with Image J. Data are expressed as mean ± SEM, analysed by Mann–Whitney. *p < 0.05.
**Figure S3:** Generation of replicative senescent SAEC. SAEC derived from healthy lungs were plated and analysed after 3 days whereas replicative senescent SAEC were plated and fed twice a week for 14 days before analysis. Cells were fixed and stained for SA‐β‐galactosidase and (a) healthy and (b) senescent SAEC were imaged by light microscopy. (c) Cell media was recovered and the concentration of PAI1 and IL‐6 was analysed by ELISA. Data are expressed as mean ± SEM, analysed by Mann Whitney. *p < 0.05.
**Figure S4:** Degradation of BCL_XL_ by BCL_XL_‐PROTAC leads to the decreased expression of senescence markers in replicative senescent SAEC. SAEC derived from healthy donors were plated and fed twice a week for 14 days, when they entered in a state of replicative senescence. Cells were then treated with 0.1 μM to 3 μM of BCL_XL_‐PROTAC for 24 to 72 h. (a) After 72 h, the expression of BCL_2_ and BCL_XL_ was measured in cell lysates by Western Blot. (b) The expression of p21^CIP1^ and p16^INK4a^ was measured in senescent SAEC by Western Blot and the production of the SASP PAI‐1 was measured in cell media by ELISA. (c) After 72 h, senescent SAEC were fixed and stained for senescence associated β‐galactosidase and imaged by light microscopy. (d) After 24–72 h, the activity of caspase 3/7 was quantified by flow cytometry. (e) After 48 h, cells were fixed and stained for DAPI (turquoise), caspase 3/7 (green), BCL_XL_ (yellow) and p21^CIP1^/p16^INK4a^ (pink) and imaged by fluorescent microscopy. Images are representative of 5 different experiments conducted on five different patients. Representative Western blots of (a) BCL_XL_ and BCL_2_ and (b) p21^CIP1^ and p16^INK4a^ expression are shown, quantified, and normalised to β‐actin. Panels (a) and (b) are derived from the same Western blot using the same samples, treatments and timepoint. Data are expressed as mean ± SEM, analysed by Kruskal–Wallis test or Two‐Way ANOVA with post hoc Sidak. *p < 0.05. **p < 0.01.
**Figure S5:** Treatment with BCL_XL_‐PROTAC leads to the activation of caspase 3/7 and decreased expression of BCL_XL_ in replicative senescent SAEC. SAEC derived from healthy donors were plated and fed twice a week for 14 days, when they entered in a state of replicative senescence. Cells were then treated with 3 μM of BCL_XL_‐PROTAC for 48 h. Cells were fixed and stained for DAPI, caspase 3/7, BCL_XL_, and p21^CIP1^/p16^INK4a^ and imaged by fluorescent microscopy. The mean intensity of caspase 3/7 (on the left) and BCL_XL_ (on the right) was analysed and quantified with Image J. Data are expressed as mean ± SEM, analysed by Mann–Whitney. **p < 0.01.
**Figure S6:** Effect of BCL_XL_ degradation by BCL_XL_‐PROTAC on p21^CIP1^ expression in COPD PCLS. PCLS derived from COPD were treated with 0.1–3 μM of BCL_XL_‐PROTAC for 48 h. Expression of p21^CIP1^ (CDKN1A) was measured in PCLS by qRT‐PCR. Data are expressed as mean ± SEM, analysed by Kruskal‐Wallis test with post hoc Dunn's. *n* = 5.
**Figure S7:** Measure of the activation of caspase 3/7 and expression of Ki‐67 in SAEC and SAF treated with BCL_XL_‐PROTAC. SAEC derived from COPD lungs were treated with 0.1–3 μM of BCL_XL_‐PROTAC. (a) After 24–72 h, cells were labelled with cell event caspase 3/7 detection reagent and the activity of caspase 3/7 was quantified by flow cytometry. (b) After 72 h, cells were fixed in 70% ethanol and stained with Ki‐67 for 30 min. The percent of cells positive for Ki‐67 was measured by flow cytometry compared to the untreated unstained condition.

## Data Availability

Data sharing not applicable to this article as no datasets were generated or analysed during the current study.
