# Supplementary material for: Clearance of Senescent Cells by BCLXL ‐PROTAC: A Novel Approach to Treat COPD?
Source: Aging Cell. 2026 Apr 14;25(4):e70487. doi: 10.1111/acel.70487 (PMC13078126; doi:10.1111/acel.70487)
Supplement: Supplementary file 1 — Figure S1: Treatment with BCLXL‐PROTAC does not modify proinflammatory chemokines and cytokines production by COPD SAEC. SAEC derived from COPD lungs were treated with 0.1 μM to 3 μM of BCLXL‐PROTAC for 72 h. The production of CXCL8 (on the left) and IL‐6 (on the right) was measured in cell media by ELISA. Data are expressed as mean ± SEM (n = 6), analysed by Kruskal–Wallis test. Figure S2: Treatment with BCLXL‐PROTAC leads to the activation of caspase 3/7 and decreased expression of BCLXL in COPD SAEC. SAEC derived from COPD lungs were treated with 3 μM of BCLXL‐PROTAC for 48 h. Cells were fixed and stained for DAPI, caspase 3/7, BCLXL and p21CIP1/p16INK4a and imaged by fluorescent microscopy. The mean intensity of caspase 3/7 (on the left) and BCLXL (on the right) was analysed and quantified with Image J. Data are expressed as mean ± SEM, analysed by Mann–Whitney. *p < 0.05. Figure S3: Generation of replicative senescent SAEC. SAEC derived from healthy lungs were plated and analysed after 3 days whereas replicative senescent SAEC were plated and fed twice a week for 14 days before analysis. Cells were fixed and stained for SA‐β‐galactosidase and (a) healthy and (b) senescent SAEC were imaged by light microscopy. (c) Cell media was recovered and the concentration of PAI1 and IL‐6 was analysed by ELISA. Data are expressed as mean ± SEM, analysed by Mann Whitney. *p < 0.05. Figure S4: Degradation of BCLXL by BCLXL‐PROTAC leads to the decreased expression of senescence markers in replicative senescent SAEC. SAEC derived from healthy donors were plated and fed twice a week for 14 days, when they entered in a state of replicative senescence. Cells were then treated with 0.1 μM to 3 μM of BCLXL‐PROTAC for 24 to 72 h. (a) After 72 h, the expression of BCL2 and BCLXL was measured in cell lysates by Western Blot. (b) The expression of p21CIP1 and p16INK4a was measured in senescent SAEC by Western Blot and the production of the SASP PAI‐1 was measured in cell media by ELISA. [file ACEL-25-e70487-s001.docx]

**Supplementary Material: Clearance of senescent cells by BCL_XL_-PROTAC: a novel approach to treat COPD?**

Justine V Devulder^1^, Peter S Fenwick^1^, Ewa Kolosionek^2^, May Al-Sahaf^3^, Patrizia Viola^3, 4^, Raphael Lemaire^5^, Neetu Razdan^5^, Hiromi Kudo^6^, Anthony Sinadinos^1^, Lina Odqvist^2^, Louise E Donnelly^1^, Peter J Barnes^1^

^1^National Heart and Lung Institute, Imperial College London, Dovehouse Street, SW3 6LY, London United Kingdom

^2^Bioscience COPD/IPF, Research and Early Development, Respiratory & Immunology, BioPharmaceuticals R&D, AstraZeneca, Gothenburg, Sweden

^3^Imperial College Healthcare NHS Trust, London, United Kingdom

^4^Division of Cancer, Department of Surgery and Cancer, Faculty of Medicine, Imperial College, London, United Kingdom

^5^Bioscience COPD/IPF, Research and Early Development, Respiratory & Immunology, BioPharmaceuticals R&D, AstraZeneca, Gaithersburg, MD, USA

^6^Section for Pathology, Division of Digestive Diseases, Department of Metabolism,

Digestion and Reproduction, Faculty of Medicine, Imperial College London, London, United Kingdom

**Corresponding author**: Justine Devulder, [j.devulder@imperial.ac.uk](mailto:j.devulder@imperial.ac.uk)

**Author Contributions**: PJB, LED, JVD designed research, JVD PSF EK HK performed research, JVD PSF AS analysed the data, RL NR LO MAS PV contributed new reagents or analytic tools, JVD wrote the paper

**
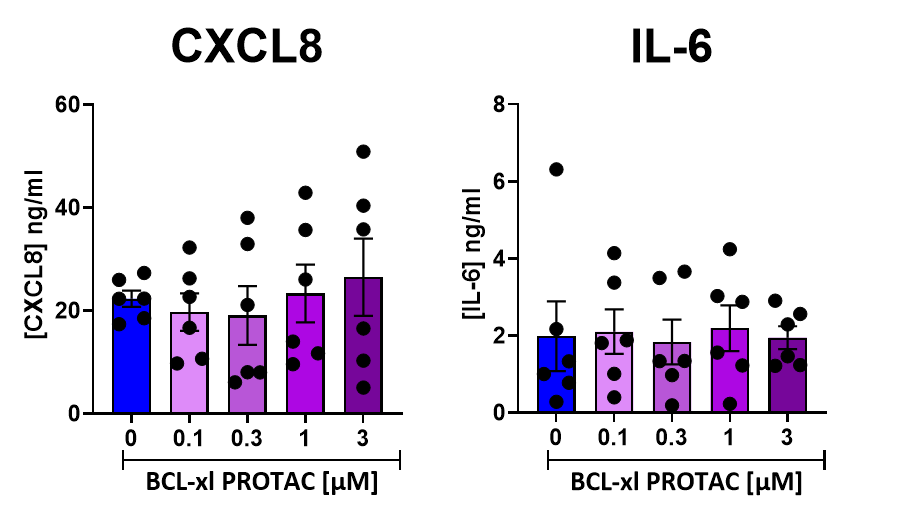
**

**Supplemental Figure 1: Treatment with BCL_XL_-PROTAC does not modify proinflammatory chemokines and cytokines production by COPD SAEC.** SAEC derived from COPD lungs were treated with 0.1μM to 3μM of BCL_XL_-PROTAC for 72 hours. The production of CXCL8 (on the left) and IL-6 (on the right) was measured in cell media by ELISA. Data are expressed as mean ± SEM (n=6), analysed by Kruskal Wallis test

**
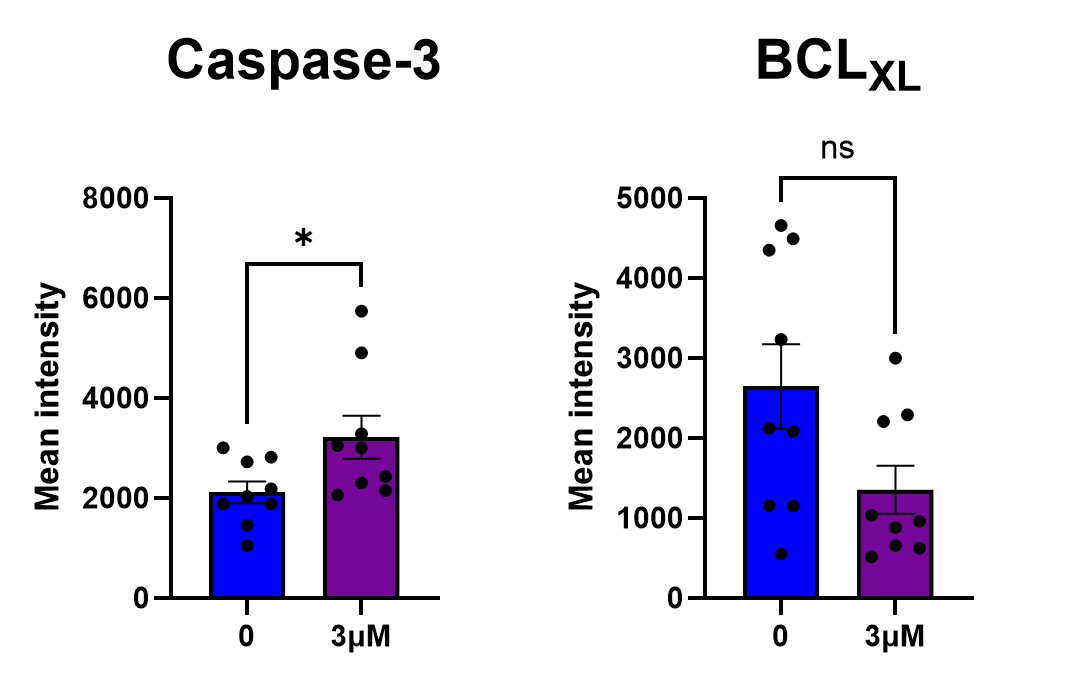
**

**Supplemental Figure 2: Treatment with BCL_XL_-PROTAC leads to the activation of caspase 3/7 and decreased expression of BCL_XL_ in COPD SAEC.** SAEC derived from COPD lungs were treated with 3μM of BCL_XL_-PROTAC for 48hours. Cells were fixed and stained for DAPI, caspase 3/7, BCL_XL_ and p21^CIP1^/p16^INK4a^ and imaged by fluorescent microscopy. The mean intensity of caspase 3/7 (on the left) and BCL_XL_ (on the right) was analysed and quantified with Image J. Data are expressed as mean ± SEM, analysed by Mann-Whitney. * p<0.05

**
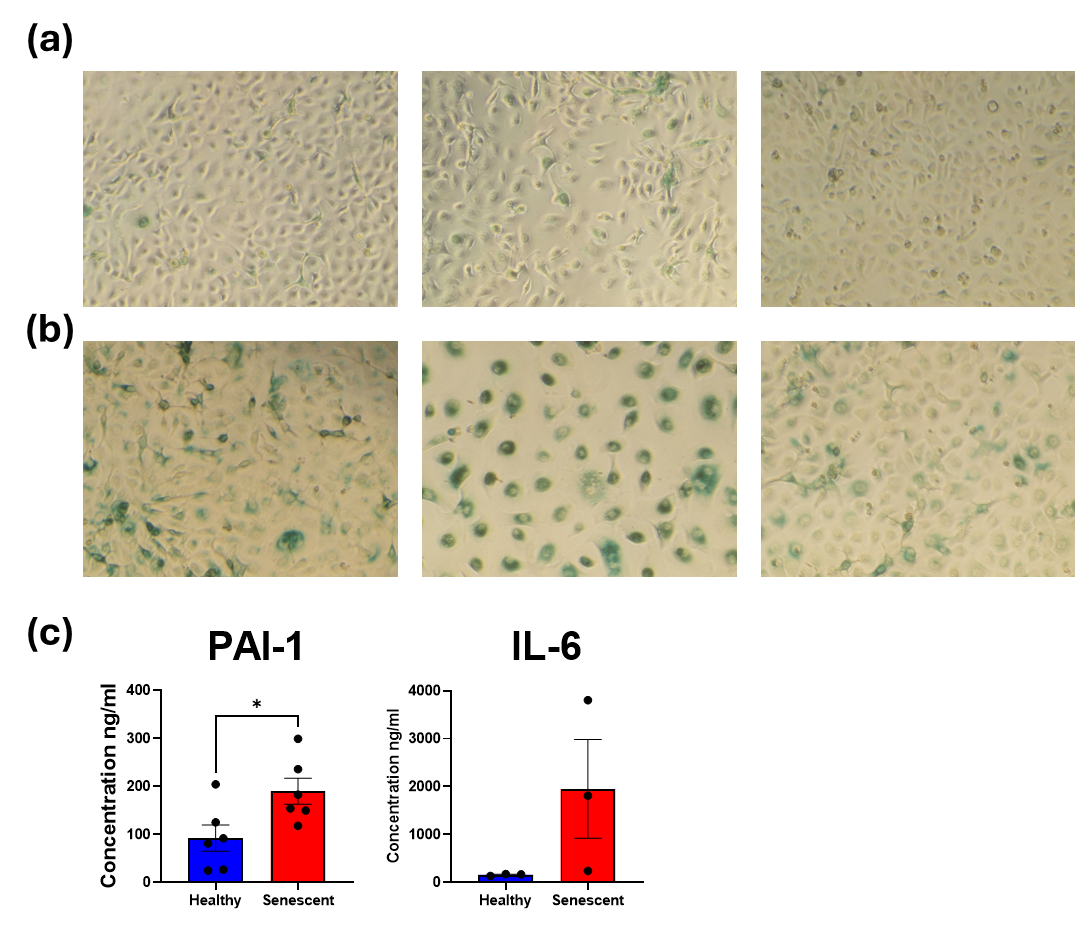
**

**Supplemental Figure 3: Generation of replicative senescent SAEC.** SAEC derived from healthy lungs were plated and analysed after 3 days whereas replicative senescent SAEC were plated and fed twice a week for 14 days before analysis. Cells were fixed and stained for SA-β-galactosidase and **a.** healthy and **b.** senescent SAEC were imaged by light microscopy. **c.** Cell media was recovered and the concentration of PAI1 and IL-6 was analysed by ELISA. Data are expressed as mean ± SEM, analysed by Mann Whitney. * p<0.05.

**
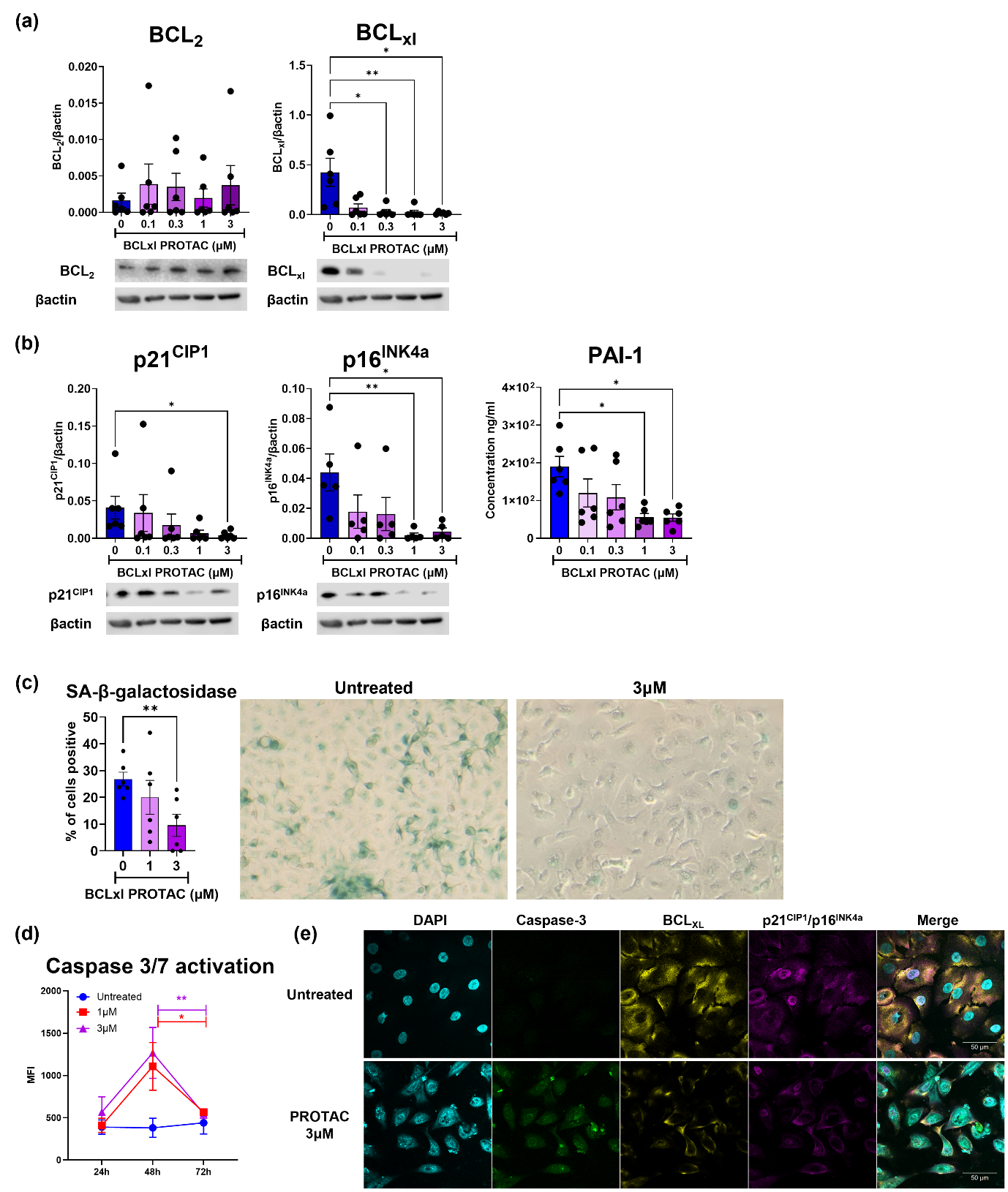
**

**Supplemental Figure 4: Degradation of BCL_XL_ by BCL_XL_-PROTAC leads to the decreased expression of senescence markers in replicative senescent SAEC.** SAEC derived from healthy donors were plated and fed twice a week for 14 days, when they become in a state of replicative senescent. Cells were then treated with 0.1μM to 3μM of BCL_XL_-PROTAC for 24 to 72 hours. **a.** After 72 hours, the expression of BCL_2_ and BCL_XL_ was measured in cell lysates by Western Blot. **b.** The expression of p21^CIP1^ and p16^INK4a^ was measured in senescent SAEC by Western Blot and the production of the SASP PAI-1 was measured in cell media by ELISA. **c.** After 72 hours, senescent SAEC were fixed and stained for senescence associated β-galactosidase and imaged by light microscopy. **d.** After 24 to 72 hours, the activity of caspase 3/7 was quantified by flow cytometry. **e.** After 48 hours, cells were fixed and stained for DAPI (turquoise), caspase 3/7 (green), BCL_XL_ (yellow) and p21^CIP1^/p16^INK4a^ (pink) and imaged by fluorescent microscopy. Images are representative of 5 different experiments conducted on 5 different patients. Representative Western blots of (a) BCL_XL_ and BCL_2_ and (b) p21^CIP1^ and p16^INK4a^ expression are shown, quantified, and normalised to β-actin. Panels (a) and (b) are derived from the same Western blot using the same samples, treatments and timepoint. Data are expressed as mean ± SEM, analysed by Kruskal Wallis test or Two-Way ANOVA with post hoc Sidak. * p<0.05. ** p<0.01.


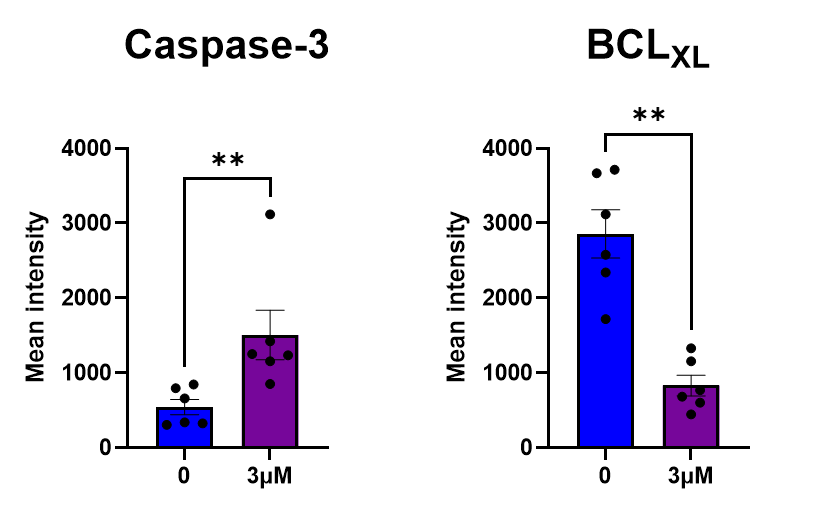


**Supplemental Figure 5: Treatment with BCL_XL_-PROTAC leads to the activation of caspase 3/7 and decreased expression of BCL_XL_ in replicative senescent SAEC.** SAEC derived from healthy donors were plated and fed twice a week for 14 days, when they become in a state of replicative senescent. Cells were then treated with 3μM of BCL_XL_-PROTAC for 48 hours. Cells were fixed and stained for DAPI, caspase 3/7, BCL_XL_ and p21^CIP1^/p16^INK4a^ and imaged by fluorescent microscopy. The mean intensity of caspase 3/7 (on the left) and BCL_XL_ (on the right) was analysed and quantified with Image J. Data are expressed as mean ± SEM, analysed by Mann-Whitney. ** p<0.01

**Supplemental Figure S6: Effect of BCL_XL_ degradation by BCL_XL_-PROTAC on p21^CIP1^ expression in COPD PCLS.** PCLS derived from COPD were treated with 0.1 to 3μM of BCL_XL_-PROTAC for 48 hours. Expression of p21^CIP1^ (CDKN1A) was measured I treated PCLS by qRT-PCR. Data are expressed as mean ± SEM, analysed by Kruskal-Wallis test with post hoc Dunn’s. n=5

**
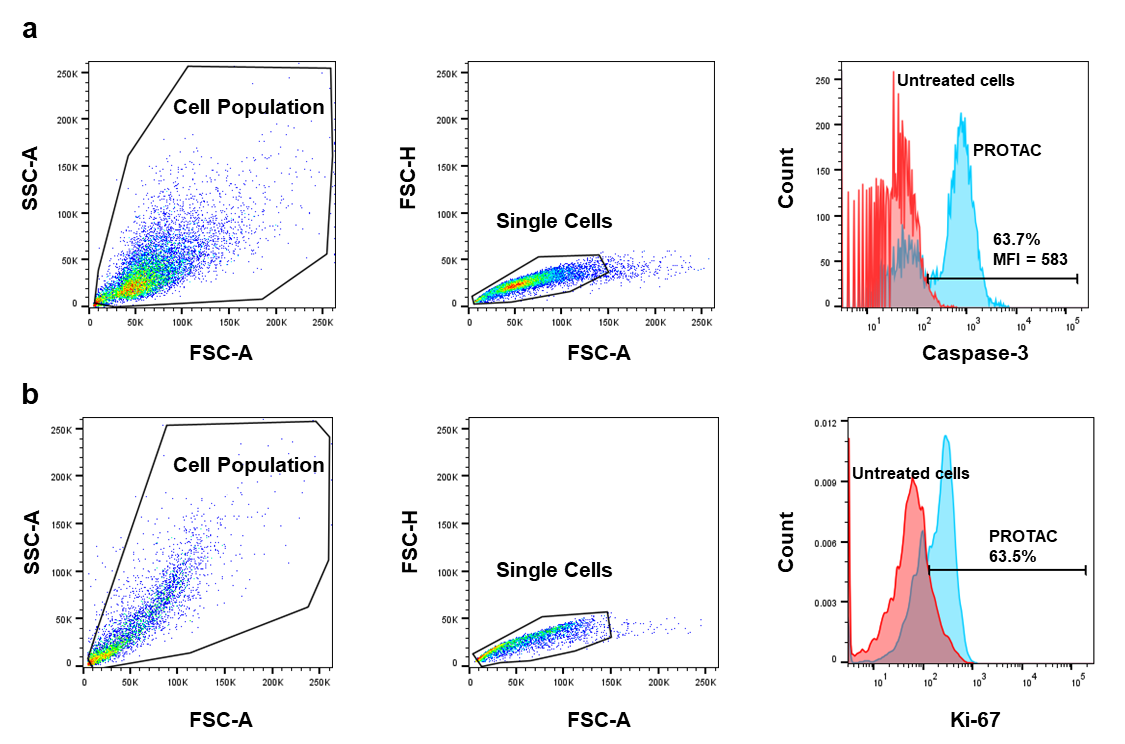
**

**Supplemental Figure 7: Measure of the activation of caspase 3/7 and expression of Ki-67 in SAEC and SAF treated with BCL_XL_-PROTAC.** SAEC derived from COPD lungs were treated with 0.1 to 3μM of BCL_XL_-PROTAC. **a.** After 24 to 72 hours, cells were labelled with cell event caspase 3/7 detection reagent and the activity of caspase 3/7 was quantified by flow cytometry. **b.** After 72 hours, cells were fixed in 70% ethanol and stained with Ki-67 for 30 min. The percent of cells positive for Ki-67 was measured by flow cytometry compared to the untreated unstained condition.
